# Psychological and Social Trajectories During Dental Treatment: A Prospective Cohort Study on Oral Health-Related Quality of Life

**DOI:** 10.3390/dj14040223

**Published:** 2026-04-09

**Authors:** Marius Moroianu, Lavinia-Alexandra Moroianu, Oana-Maria Isailă, Cătălin Pleșea-Condratovici, Simona-Dana Mitincu-Caramfil, Mădălina Nicoleta Matei

**Affiliations:** 1 Faculty of Medicine and Pharmacy, Research Centre in the Medical-Pharmaceutical Field, Dunarea de Jos University of Galați, 800201 Galați, Romania; moroianu.g.marius@gmail.com (M.M.); catalin.plesea@ugal.ro (C.P.-C.); simona_caramfil@yahoo.com (S.-D.M.-C.); madalina.matei@gmail.com (M.N.M.); 2 Dental Office, MORODENT OPTIMUM SRL, 1 Movilei Street, 800121 Galați, Romania; 3 Clinical Hospital of Psychiatry “Elisabeta Doamna”, 290 Traian Street, 800179 Galați, Romania; 4 Department of Legal Medicine and Bioethics, Faculty of Dental Medicine, “Carol Davila University” of Medicine and Pharmacy, 020021 Bucharest, Romania

**Keywords:** dental treatment, quality of life, psychosocial trajectories, prospective cohort, patient-reported outcomes, questionnaire, generalized estimating equations, McNemar test

## Abstract

**Background**: Patients undergoing dental treatment often experience psychological distress and social discomfort, yet longitudinal data on these changes are limited. Existing studies rely on cross-sectional designs or lengthy tools, reducing feasibility in routine practice. This study explored psychological and social trajectories during dental care, highlighting challenges and implications for patient wellbeing and care delivery. **Methods**: A prospective cohort study with repeated measures across three dental visits (V1–V3) was conducted. Participants completed a 21-item binary (yes/no) questionnaire assessing psychological (Q1–Q6) and social dimensions (Q7–Q14 at all visits; extended social domain Q7–Q21 at V2–V3). Composite scores were calculated, and longitudinal changes were analyzed using generalized estimating equations or mixed-effects models. Item-level trajectories were examined with multiple comparison adjustments. **Results**: Of 120 enrolled patients, 100 completed all visits. Psychological well-being consistently improved, while social outcomes showed more complex, domain-specific patterns. Item-level analyses revealed gains in appearance and satisfaction, whereas stigma, fear, and social integration remained relatively stable, underscoring the need to monitor multiple psychosocial dimensions. **Conclusions**: Psychosocial monitoring during dental care is feasible and potentially beneficial. The 21-item questionnaire was practical and well-accepted, with composite scores serving as simple indicators for tracking patient wellbeing and supporting a holistic, patient-centered approach. Further validation in larger and more diverse populations is needed.

## 1. Introduction

Oral health is increasingly recognized as a core component of overall health and well-being, with downstream implications that extend beyond symptoms and function to include psychological status, social participation, and broader quality-of-life outcomes [1,2,3,4]. Dental treatment is conducted within a complex biological and procedural environment, where restorative and orthodontic metallic materials are exposed to dynamic salivary conditions and acidic challenges that may influence treatment characteristics and patient experience. Experimental evidence shows that commonly used dental alloys exhibit variable corrosion behavior in salivary solutions, particularly in the presence of lactic acid, underscoring the multifactorial nature of dental care contexts [5]. Contemporary global health agendas emphasize that oral diseases remain highly prevalent and, when untreated or poorly controlled, may act as chronic stressors that compound existing vulnerabilities and widen inequities [1,2,3]. Conceptually, these links are best captured by biopsychosocial frameworks, which posit that clinical outcomes emerge from dynamic interactions among biological processes, cognitive and affective responses, behaviors, and social context [6,7,8]. In dentistry, this perspective is particularly salient because many treatments are performed in proximity to perceived threats (such as pain, loss of control, or anticipated negative outcomes), making patient-reported psychological and social responses central to the clinical experience.

Dental care is also a well-established trigger for anxiety and fear, which may influence both care-seeking behavior and the perceived burden of treatment [9,10,11,12,13,14]. Dental anxiety has been linked to broader psychopathology and comorbid psychiatric symptoms, underscoring that distress during dental treatment is not merely situational but may reflect a broader vulnerability profile [9,10,11,12]. Moreover, anxiety and pain mutually reinforce one another across procedures and treatment stages, with implications for symptom management, adherence, and satisfaction [13]. These mechanisms are clinically relevant across age groups, as dental anxiety is commonly reported in adolescents and adults alike, with potential developmental and experiential correlates (including prior distressing or traumatic experiences and general psychological status) [14,15,16]. Valid, feasible measures of dental anxiety and related constructs are therefore necessary both for clinical risk stratification and for understanding how distress evolves during treatment [17,18].

Evidence from other medical fields indicates that psychological distress is not static but evolves dynamically across disease and treatment trajectories. In oncology, systematic evidence shows that distress, coping responses, and psychosocial outcomes vary substantially over time and across therapeutic phases, supporting the need for repeated, stage-specific assessment rather than single cross-sectional evaluations [19].

Beyond symptom perception, dental anxiety may shape patient–provider interactions, engagement, and decision-making, potentially leading to postponed care, more complex presentations, and increased psychosocial burden [11,20,21,22,23]. While a range of behavioral and communication strategies has been proposed to mitigate dental anxiety in routine practice, implementation remains heterogeneous and often depends on time constraints, training, and the availability of structured screening tools [20,21]. More recently, integrative models have emphasized that dental fear and anxiety should be understood alongside broader sensory–emotional processing, general anxiety tendencies, and oral health behaviors, reinforcing the need to assess multiple psychosocial dimensions rather than relying on a single-domain measure [22]. Patients’ own perceptions of oral healthcare experiences are likewise central to designing psychologically informed pathways that reduce distress and improve engagement [23].

Beyond the reduction in distress, psychological well-being is increasingly conceptualized in terms of positive resources such as optimism and positive affect, which may buffer stress and facilitate adaptation to medical challenges. Evidence suggests that these resources are context-dependent and can fluctuate across major life or health-related transitions, reinforcing the need for repeated assessment over time [24].

Importantly, psychosocial burden during dental treatment is not limited to internal distress. Oral conditions and their treatment can affect social functioning and identity through visible changes, perceived stigma, and limitations in social participation [25]. This is consistent with broader evidence that oral health status is strongly linked to health-related quality of life (HRQoL) and oral health-related quality of life (OHRQoL), which capture functional, emotional, and social impacts that matter to patients [4,26,27,28,29]. OHRQoL research has shown clinically meaningful changes following restorative and prosthetic interventions, yet these outcomes are often evaluated as pre–post snapshots rather than as trajectories across distinct stages of care [27,28,29]. Social resources may also moderate distress and quality-of-life outcomes: social support, self-efficacy, and the structure of support networks have been associated with better OHRQoL and may buffer the adverse effects of stressors and discrimination [30,31]. Taken together, these findings suggest that dental treatment can initiate coupled psychological and social trajectories that are likely to vary across treatment phases and between individuals.

Although conceptually related to OHRQoL, the present instrument was designed to capture short-term psychosocial trajectories rather than to operationalize a full QoL construct.

However, there remains a practical gap in the literature: prospective, multi-visit studies that map within-person psychosocial trajectories across key stages of dental treatment are scarce, and clinical settings often require brief tools that are feasible for repeated administration [26,27,28,29,32,33]. In line with patient-reported outcome (PRO) standards emphasizing content validity, interpretability, and feasibility in the target context [32,33], we developed an original, brief, binary-response questionnaire intended to be used repeatedly during routine care. The present study aimed to prospectively evaluate psychological and social trajectories during dental treatment using a brief patient-reported questionnaire administered across three clinical visits.

## 2. Materials and Methods

### 2.1. Study Design and Reporting

This study was designed as a prospective observational longitudinal cohort conducted in a real-world dental care setting at a single clinical center. Participants were evaluated during three predefined clinical visits (V1–V3), corresponding to the pre-treatment phase, the active course of dental treatment, and the post-treatment follow-up. The design enabled the assessment of psychosocial changes occurring throughout the dental care process. Each participant served as their own control, allowing within-subject comparisons between baseline and subsequent follow-up assessments, without the inclusion of a parallel control group. In PIC(O) terms, the population consisted of adults undergoing dental treatment, the exposure corresponded to the routine sequence of dental procedures between baseline and final follow-up, the comparison involved longitudinal self-comparison of baseline versus follow-up measurements, and the outcomes included changes in binary questionnaire responses and composite psychosocial scores. The study design and reporting followed the STROBE recommendations for cohort studies to ensure transparency and methodological rigor.

The full original version of the questionnaire is provided in Appendix A.

### 2.2. Setting and Timeline

The research was conducted at a single clinical site—a private dental medicine clinic—in an urban area of southeastern Romania (clinic identity is de-identified). All study visits took place in the dental clinic setting, integrated into each participant’s routine treatment appointments. The recruitment and follow-up period spanned from 10 January 2022 to 2 October 2023. Participants were enrolled and evaluated on a rolling basis during this interval as they initiated and progressed through their dental treatment plans. Each participant had three scheduled study visits: a baseline visits before or at the start of treatment (V1), an intermediate visit during the course of treatment (V2), and a post-treatment visit after completion of all planned dental interventions (V3). The timing of V2 and V3 was aligned with the individual’s treatment milestones rather than fixed calendar intervals, to correspond with mid-treatment and end-of-treatment points for each patient. This flexible scheduling ensured the study assessments coincided with meaningful stages of the therapeutic trajectory while maintaining a consistent overall structure of three visits for every participant.

### 2.3. Participants

We used consecutive sampling, inviting all eligible patients from the clinic to participate in the study as they began a new course of dental treatment. The target sample included adult patients (aged ≥18 years) who were initiating a dental treatment plan and were expected to be available for all three planned visits (baseline, mid-treatment, and post-treatment). Key inclusion criteria were the ability to understand the questionnaire and provide informed consent, and a commitment to return for the scheduled follow-up visits. Patients were excluded if they could not complete at least the baseline evaluation, were unlikely to complete the full V1–V3 sequence (for example, due to foreseeable relocation or unwillingness to return) or had any condition that would lead to incomplete essential data. All participants provided written informed consent before enrollment, in accordance with ethical requirements. Patients were recruited consecutively among individuals presenting to the dental clinic for consultation or treatment. The clinical reasons for presentation could include routine dental evaluation, dental pathology requiring treatment, or urgent dental symptoms. However, the specific clinical reason for each visit was not systematically categorized in the present analysis.

A total of 120 participants were enrolled and assessed at Visit 1 (initial baseline). Between Visit 1 and Visit 3, 20 individuals were lost to follow-up or excluded from analysis due to unavailability for scheduled follow-ups, withdrawal of consent, or missing essential data. Because the analytical dataset was restricted to participants who completed all three visits, detailed demographic and questionnaire data were not systematically retained for individuals who did not continue beyond the baseline assessment. Therefore, a formal comparison between completers and non-completers was not feasible. However, participant flow and retention are transparently reported in the study description. This resulted in N = 100 participants (83.3% of those enrolled) who completed all three visits and were included in the longitudinal analyses. These 100 participants ranged in age from 18 to 79 years, with a mean age in mid-adulthood (the cohort included both young adults and seniors). The final sample was 65% female and urban-residing (approximately 87% from urban areas), reflecting the clinic’s patient population. All participants were managing various dental conditions requiring multi-step treatment (e.g., restorative, surgical, and/or prosthetic interventions), but no specific diagnosis-based exclusions were applied aside from the general criteria above. Participant flow and retention are illustrated in a STROBE flow diagram (adapted from the thesis) showing 120 at baseline, 20 losses, and 100 completers analyzed longitudinally.

### 2.4. Procedures at Visits

Each participant underwent three standardized study visits integrated with their dental treatment timeline:

**Visit 1 (Baseline, Pre-intervention):** At the initial visit—before or at the start of any new dental interventions—participants completed the psychosocial questionnaire (administering items Q1–Q14 at this stage). This baseline questionnaire captured the patient’s psychosocial status before treatment. Additionally, a brief 4-item anxiety/depression screener was administered at presentation as part of the clinical protocol. The screener quickly identified acute distress (for patient safety and triage) and was separate from the main 21-item instrument. All baseline data, including questionnaire responses and relevant clinical information, were recorded in the participant’s file. Dental treatment (e.g., evaluation or urgent care) proceeded according to standard practice after the questionnaires, with no study interventions applied (observational design).

**Visit 2 (Intermediate, Mid-treatment):** Participants returned for a second evaluation at an intermediate point in their treatment course (for example, after initial procedures had been completed but before final prosthetic or restorative work). At V2, the full set of 21 questionnaire items was administered. This included the original 14 baseline items (repeated to track changes) plus 7 additional items that specifically inquired about the patient’s perceived improvements and satisfaction following dental treatment up to that point. The timing of V2 coincided with a natural pause or milestone in the treatment (e.g., after healing from a surgery or between multi-step procedures), ensuring that the participant could reflect on any initial outcomes. The questionnaire was completed in the clinic (in an undisclosed area of the waiting room or operator before the dental procedure of that visit). Research staff ensured the form was fully completed and clarified any patient questions. The patient then continued with the planned dental treatments for that visit as usual.

**Visit 3 (Post-intervention, outcome):** The third visit occurred after the patient’s planned dental treatments were completed (i.e., post-intervention). It was typically scheduled for the final checkup or shortly after the last treatment session. At V3, the 21-item questionnaire was administered again (identical to V2) to assess the participant’s psychosocial status after treatment completion. The focus at V3 was on capturing end-of-treatment outcomes—for instance, whether the patient felt happier, less stigmatized, or better socially integrated after resolving their dental problems. Following questionnaire completion, no final clinical assessments or routine dental follow-up care were provided. V3 marked the conclusion of study participation.

All questionnaire forms were collected by the study personnel at each visit and checked for completeness in real time. The administration was conducted in the participant’s native language (Romanian) in an easy-to-understand format, and clarifications were provided as needed, though most participants self-reported their answers. Importantly, the 4-item safety screener for anxiety/depression was also applied at baseline (and repeated at later visits if indicated by protocol) to identify any severe psychological distress promptly. Participants who endorsed any item suggesting severe depression or suicidal ideation (e.g., thoughts of death) were flagged, and the dental team had a protocol to provide or refer for appropriate psychological support (this safety protocol was defined a priori in an ethical annex). Aside from completing questionnaires, no experimental procedures were performed—all other care received by participants was standard-of-care dental treatment determined by their clinicians.

### 2.5. Instrument: Psychosocial Questionnaire

**Development:** The primary measurement tool was an original 21-item binary-response questionnaire developed by the research team for this study. This instrument was specifically designed to capture the psychosocial state of dental patients before, during, and after treatment, in the context of oral health-related anxiety and depression. The content of the questionnaire was informed by literature on oral health-related quality of life, dental anxiety, and psychosocial impacts of dental disorders, as well as clinical experience with patients reporting emotional and social challenges due to poor oral health.

The questionnaire was subjected to a preliminary face validity assessment by a small expert panel (*n* = 4) before its use in the cohort study. The panel included clinicians and researchers with relevant expertise in dentistry, psychiatry, and medical research methodology. Experts reviewed the proposed items to evaluate conceptual clarity, clinical relevance, and comprehensibility for patients undergoing dental treatment. Their feedback led to minor wording adjustments and refinement of several items to improve clarity and interpretability.

A formal quantitative content validity index (CVI) was not calculated, as the review process was intended primarily to ensure face validity and practical usability of the instrument in a clinical setting.

The questionnaire was subsequently pilot-tested in a small group of dental patients (*n* = 10) before the start of the study to confirm feasibility, comprehension, and completion time. Based on patient feedback, minor wording adjustments were implemented, and the finalized 21-item questionnaire was then applied in the prospective cohort.

The final questionnaire consisted of concise statements (items) to which the patient responds “Yes” or “No,” reflecting whether they experience a particular feeling or situation.

**Content:** The 21 items cover two broad domains: a **Psychological domain** and a **Social domain**, with some items only applicable after treatment has begun. In the **Psychological domain (Q1–Q6)**, items assess symptoms of anxiety and depression related to dental health. Examples include feeling anxious or panicked about one’s dental appearance or oral health (Q1), feeling sad or depressed because of the dental appearance (Q2), loss of appetite or weight due to dental problems (Q3, Q4), disturbed sleep with nightmares about oral health (Q5), and even thoughts of death associated with stigma from dental issues (Q6). These items gauge the mental health burden (stress, low mood, even suicidal ideation) that dental problems can impose. The **Social domain** items capture the impact of dental issues on social functioning, self-image, and support. At baseline, the Social domain included 8 items (Q7–Q14): for instance, satisfaction with one’s facial/dental appearance (Q7, Q8), feelings of inferiority or stigma due to poor teeth (Q9, Q10), having plans (hopefulness) despite dental issues (Q11), difficulty integrating into community because of dental problems (Q12), perceived support from partner/family in dealing with dental problems (Q13), and fear of upcoming dental procedures (dental anxiety) (Q14).

In addition, 7 supplemental items (Q15–Q21) were included in the questionnaire to be answered only at the follow-up visits (V2 and V3) once the patient had undergone some treatment. These items specifically probe positive outcomes or improvements after dental treatment: e.g., satisfaction with the dental treatments received so far (Q15), satisfaction with the price/quality ratio of the treatment (Q16), feeling happier after resolving dental problems (Q17), perceiving less stigma after treatment (Q18), improved eating/diet after treatment (Q19), better social integration after treatment (Q20), and improved sleep after the recent treatments (Q21). By design, these post-treatment items could not be meaningfully answered at baseline (when no treatment had occurred), but at V2 and V3, they capture any psychosocial gains resulting from the dental interventions.

**Response Coding and Scoring:** All questionnaire items were binary (Yes/No answers). For analysis, responses were coded as 0 or 1 in such a way that higher scores indicate a more favorable outcome on each item. In practice, this meant that items phrased negatively (problems or symptoms) were reverse-coded, while items phrased positively were coded at face value. For example, a “Yes” (DA) answer to “Do you feel anxious about your dental appearance?” (a negative symptom) was coded 0 (unfavorable), whereas “No” was coded 1 (favorable, indicating absence of anxiety). Conversely, for a positively phrased item like “Do you feel happier after treatment?”, “Yes” was coded 1 (favorable). In summary, each item’s favorable response (whether that be “No” for a negative symptom or “Yes” for a positive outcome) was scored as 1, and an unfavorable response as 0. This recording ensured that for every item and any aggregated score, a higher value consistently reflected better psychosocial status (less stress, or more satisfaction).

The composite indices were designed to reflect overall psychosocial trajectories during dental treatment rather than isolated symptom domains. Baseline items capture psychosocial burden associated with dental problems, while additional follow-up items assess perceived improvements after treatment. Combining these elements allows the composite score to represent the transition from psychosocial deficit toward improvement across the treatment process.

The questionnaire yields several derived scores/outcomes (see Section 4.6 below) based on grouping the items: a total composite score and subscale scores for the Psychological and Social domains. Scoring rules were defined a priori in the analysis plan. In brief, subscale scores were calculated as the proportion of items answered favorably in that domain, i.e., the mean of the item scores (ranging from 0 to 1). For instance, a Psychological score was computed as the mean of the six Q1–Q6 binary scores, and a Social score as the mean of the Social-domain item scores. At V1, the Social score was based on Q7–Q14 (8 items), whereas at V2 and V3 it was based on Q7–Q21 (15 items) since the domain was extended with post-treatment items. For longitudinal comparisons, we also examined a “core” social subscale (Q7–Q14 only) common to all visits for consistency. A global composite score (overall proportion of all favorable responses) was likewise computed per visit as an indicator of overall psychosocial well-being related to oral health. If a participant skipped an item or it was not applicable, a missing data rule was applied (see Section 2.7) to decide whether the score could be calculated from the remaining items.

**Reliability:** The instrument demonstrated acceptable internal consistency for an exploratory tool of this nature. We assessed reliability using the Kuder–Richardson Formula 20 (KR-20), which is equivalent to Cronbach’s alpha for dichotomous items. At baseline, the 8-item Social subscale already showed good internal consistency (KR-20 ≈ 0.77). The 6-item Psychological subscale was lower (KR-20 in the 0.58–0.60 range at V1), reflecting the diverse symptoms captured. By the intermediate visit (V2), after all 21 items were introduced, the total scale reliability was high (KR-20 ≈ 0.87), indicating high internal consistency. The Social domain remained more internally consistent than the Psychological domain at each time point, which is plausible given that the social items encompass more behaviorally and functionally related content that tends to co-vary, whereas the anxiety/depression items may be more variable across individuals. At the final visit (V3), reliability coefficients slightly dipped (total scale KR-20 around 0.77) as variability in responses decreased post-treatment (a common effect when everyone’s outcomes improve and responses become more uniformly positive). Overall, these findings suggest the questionnaire had adequate reliability for group-level analysis in this observational study, with consistency improving when the full range of items was considered.

Because the questionnaire represents a newly developed instrument, a minimal clinically important difference (MCID) has not yet been established. Therefore, the present analysis focused on relative longitudinal changes in composite scores and item-level responses rather than predefined clinical thresholds.

### 2.6. Outcomes

The primary outcomes for this study were composite psychosocial scores derived from the questionnaire at each visit, along with the change in these scores over time. The principal outcome measure was defined as the proportion of favorable responses (i.e., the fraction of questionnaire items answered in the positive direction) per participant per visit. This could be interpreted as an overall psychosocial score ranging from 0 (all answers indicate psychosocial distress or dissatisfaction) to 1 (all answers indicate a positive psychosocial state). In addition to this global composite, we examined two domain-specific composite scores:

**Psychological Score:** the proportion of favorable responses to the 6 psychological items Q1–Q6 (covering anxiety, depression, etc.). This reflects the level of psychological distress vs. well-being related to oral health. A higher Psychic score means fewer anxiety/depression symptoms and thus a better psychological state.

**Social Score:** the proportion of favorable responses to the social-impact items. At baseline, this Social composite was based on Q7–Q14 (8 items focusing on social and self-image impacts). At the follow-up visits (V2, V3), the Social composite was expanded to include Q7–Q21 (15 items, incorporating the additional questions on post-treatment improvements). A higher Social score indicates better social functioning and satisfaction (e.g., less stigma, more social confidence and support). For comparisons across all three time points, we also evaluated the Social domain using only the core 8 baseline items (Q7–Q14) so that the same content was compared from V1 to V3.

All these outcomes are expressed on a continuum from 0 to 1 (or equivalently 0% to 100% favorable responses) for ease of interpretation. Higher scores correspond to better outcomes—for example, a higher Psychological score means the patient reported fewer negative emotional symptoms, and a higher Social score means the patient felt more socially confident and supported. This directionality was ensured by the recoding of item responses (Section 4.5) and was confirmed the Friedman test and Kendall’s W effect size, which indicated that a higher composite score corresponds to a more favorable psychosocial status for all scales.

In addition to the composite scores, the change in individual item responses between visits was considered a secondary outcome for fine-grained analysis. In particular, we were interested in the transition of answers (Yes → No or No → Yes) from baseline to post-treatment on key items like “feeling stigmatized” or “having plans”. These item-level changes (improvements or deteriorations) provide qualitative insight into which specific psychosocial aspects improved significantly with treatment. We thus treated each questionnaire item’s yes/no response at different time points as paired binary outcomes to analyze statistically (see Section 2.8). Finally, the study also recorded basic dental health outcomes (the completion of planned dental treatment for each participant), but the primary focus remained on psychosocial outcomes rather than clinical dental indices.

### 2.7. Data Management and Missing Data

All study data were handled with rigorous data management practices to ensure accuracy, confidentiality, and integrity. Data Collection and Entry: At each visit, the completed questionnaires were collected by the investigators and immediately double-checked for completeness. Responses were then entered into secure electronic databases (with appropriate coding) parallel with the patient’s clinical record. Each participant was assigned a unique study ID, and data were recorded under these pseudonymous IDs. No direct personal identifiers (name, address, national ID number) were stored with the research data. The main list linking participant identities to their study IDs was kept separately by the principal investigator under lock and key, in accordance with data protection regulations. This ensured that analysis datasets were fully de-identified. The study complied with GDPR and relevant national data protection laws throughout data handling.

**Quality Control:** The research team implemented standardized procedures for data entry and quality assurance. All staff involved in data collection were trained using the same protocol for administering the questionnaire and recording answers to minimize inter-observer variability. Data entry was verified by cross-checking against paper forms, and any discrepancies were resolved by consulting the source documents. The dataset was audited internally for logical consistency (e.g., verifying that skip patterns and eligibility criteria were respected, and that no out-of-range values were present). Before locking the final analysis dataset, internal consistency checks were performed—for example, ensuring that the number of “Yes”/“No” answers per questionnaire matched the expected totals, and that no duplicate entries existed. The electronic records included time stamps and version control. An audit trail was maintained for any data cleaning steps, with a data dictionary documenting all variable coding and any transformations. The final dataset was “frozen” (no further changes allowed) before analysis and archived with a digital signature (SHA-256 checksum) to guarantee integrity. Overall, these measures ensured an elevated level of data integrity and reproducibility in line with Good Clinical Practice for data management.

**Missing Data and Retention:** Thanks to the careful screening of participants and follow-up efforts, missing data were very limited in this study. As noted, 83% of enrolled participants completed all visits, providing a strong retention rate. The 20 participants who did not complete the study were removed from the longitudinal analyses; their baseline data were not carried forward except for describing initial sample characteristics. Among the 100 completers, item-level missing responses on the questionnaires were rare. If a participant left an occasional questionnaire item blank or answered, “I don’t know” (an option discouraged but recorded if it occurred), the protocol’s a priori missing data rules were applied: we allowed calculation of a composite score so long as the missing responses were minimal. Specifically, a Psychological or Social subscale score could be computed for a given visit if at most one item in that subscale was unanswered at baseline (for the 6-item Psych or 8-item Social scales) or at most two items were missing in the 15-item Social scale at V2/V3. In such cases, the composite was calculated as the mean of the answered items. If missing responses exceeded these thresholds, that subscale score for that visit was considered missing for that participant (this was an extremely uncommon scenario). These rules were implemented to preserve data from participants who might skip one sensitive question but otherwise provide complete information. The analytic dataset had complete data for the primary outcomes; the incidence of any missing item in the 21-item set was very low, and no pattern of missingness suggesting bias was observed.

**Missing Data Strategy:** We planned a conservative approach to handle any potential missing data in outcomes. First, we assessed whether the missing data were random. A Little’s MCAR test was performed to check if any item-level missingness was completely at random. If the data had not been consistent with MCAR (and if more substantial missingness had occurred), the analysis plan provided for a multiple imputation strategy under the assumption of missing at random (MAR). We specified the use of Multiple Imputation by Chained Equations (MICE) with *m* = 20 imputed datasets, including relevant auxiliary variables (such as age, sex, baseline scores) to inform the imputation model. The pooled estimates across imputations would then be combined using Rubin’s rules. In parallel, all primary analyses were also prespecified to be run on a complete-case basis for comparison (excluding any cases with missing outcome data). In practice, however, the need for imputation did not materialize because data completeness was almost perfect among the analyzable cohort, so the primary results are based on observed data. We did perform sensitivity analyses with complete-case vs. available-case data and found no meaningful differences in outcomes or conclusions. Thus, the high retention and minimal missing data ensured that the results have strong internal validity without reliance on imputation.

### 2.8. Statistical Analysis

All data were analyzed according to a pre-specified statistical analysis plan, focusing on describing the psychosocial measures at each time point and testing the significance of changes over time. **Descriptive statistics** were used to summarize participant characteristics and outcome variables at baseline and follow-ups. We calculated means and standard deviations (or medians and interquartile ranges, if non-normal) for continuous composite scores, and frequencies/percentages for categorical variables and individual item responses. Baseline and follow-up composite score distributions were visualized to assess normality and floor/ceiling effects. Internal consistency of the questionnaire at each visit was quantified by the Kuder–Richardson-20 coefficient (with 95% confidence intervals via the Feldt method or bootstrap), as noted in Section 4.5. For example, KR-20 values at each visit were reported to evaluate reliability trends over time (these results were described in Section 4.5 but also statistically calculated here).

**Longitudinal Models:** The primary analytic focus was on the change in composite **scores** (Psychological and Social) from V1 to V2 to V3. To rigorously assess these longitudinal changes, we employed Generalized Estimating Equations (GEE) models for repeated measures. GEE is a population-averaged approach that accounts for within-subject correlation over time. We specified an identity link function with a normal error distribution for the composite scores (treating the proportion scores as continuous outcomes). An exchangeable working correlation structure was assumed (meaning we modelled that measurements from the same individual are equally correlated), and robust (sandwich) standard errors were used to ensure valid inference even if the correlation structure was mis-specified. Each GEE model included **Visit (time point)** as a categorical predictor (to estimate differences from baseline at V2 and V3) and was adjusted for several a priori covariates. Specifically, we adjusted for baseline age (as a categorical age group, since the age range was broad), sex, and residential environment (urban vs. rural), as these factors might influence psychosocial outcomes. We also adjusted for the type of dental intervention the patient underwent during the study, categorized into major procedure groups (e.g., hygiene, fillings, endodontic therapy, extractions, prosthodontic work, other). The inclusion of intervention type was to account for the possibility that more complex or invasive treatments might have different psychosocial impact trajectories than simpler treatments. In the GEE analysis, parameter estimates were obtained for the mean change in Social score and Psychic score at V2 and V3 compared to V1, with 95% confidence intervals and *p*-values testing whether these changes were significantly different from zero. The GEE results thus provided adjusted estimates of improvement between visits.

In addition to GEE, we performed a complementary analysis using a mixed-effects regression approach to ensure the robustness of results. Specifically, we ran linear regression models equivalent to repeated-measures ANOVA (treating participant as a random effect); however, given the small number of time points and our interest in population-average effects, this was simplified to an OLS model with cluster-robust standard errors on participant ID (which is algebraically similar to a mixed model with random intercepts). This approach (OLS with Huber–White robust SE, sometimes called LPM for “linear probability model” since our outcomes are proportions) yielded identical direction and magnitude of effects as the GEE models. The concordance between the GEE and the OLS/mixed-model framework confirmed that our findings were not an artifact of the modeling method. We also conducted a non-parametric repeated measures test (Friedman test) for overall change in the composite scores across the three visits, with Kendall’s W reported as an effect size measure for the within-subject change, to double-check the significance of improvement without assuming normality. This also supported the presence of meaningful change (these results are described in Section 3 rather than here).

**Item-Level and Secondary Analyses:** To analyze changes in individual item responses over time, we used McNemar’s test for paired binary data. For each questionnaire item, we compared the proportion of “Yes” responses at baseline vs. follow-up (or vice versa) among the same individuals. McNemar’s tests were run primarily between V1 and V3 (pre vs. post) to identify which specific psychosocial indicators showed statistically significant improvement. We applied a continuity correction (Haldane–Anscombe adjustment) for items where discordant pairs were sparse, which can stabilize the odds ratio estimate in those cases. Because multiple items (21 tests) were examined, we controlled the family-wise false discovery rate using the Benjamini–Hochberg procedure. An adjusted *p*-value (q-value) was computed for each item’s McNemar test to determine significance after FDR correction. This way, we could identify which items improved significantly beyond chance, while limiting false-positive findings from multiple comparisons. As expected, the composite score analysis (which aggregates across items) provided the primary evidence of improvement, and item-level tests were mostly used to illustrate the pattern of changes. We interpret item-level results cautiously and qualitatively (given some items had rare changes, leading to wide confidence intervals).

**Sensitivity Analyses:** We conducted several sensitivity and subgroup analyses to test the robustness of our conclusions. First, a complete-case analysis was performed in parallel—i.e., restricting to the subset of participants who had no missing data at any visit (which in our case was the same 100 participants, since missingness was negligible). This produced the same direction and significance of results as the main analysis (no change in conclusions). Second, we tried alternative correlation structures in the GEE (such as unstructured and autoregressive) to ensure our results were not sensitive to the working correlation assumption; the estimates of change remained identical, confirming that the improvements observed are robust. Third, we examined whether the results differed by key subgroups: we added interaction terms to the models to see if improvement from V1 to V3 was different in women vs. men, or younger vs. older participants, etc. No significant interactions were found with sex or age, indicating the positive trend was present across these groups (though statistical power for interactions was limited). We also stratified the analysis by broad intervention type (e.g., comparing those who had primarily surgical interventions vs. primarily restorative) and found the trend of psychosocial improvement held in all subgroups, though magnitudes varied slightly (with, for example, those receiving prosthetic rehabilitations showing some of the largest psychological gains, consistent with their baseline deficits). Additionally, a leave-one-out analysis was done: we re-ran the main models, omitting each participant one at a time, to check that no single individual was unduly influencing the results. This jackknife procedure showed stable results, i.e., removal of any given case did not appreciably alter the overall effect estimates. All these sensitivity checks converged on the same finding: a substantial and robust improvement in psychosocial scores from pre- to post-treatment, not driven by outliers or modeling choices. Finally, we developed an exploratory logistic regression model to predict the likelihood of a “favorable response” on each item at follow-up based on baseline status and covariates, but those analyses are beyond the scope of this Methods section (they were used to generate hypotheses for future research).

**Statistical significance** was set at a two-tailed alpha of 0.05 for primary endpoints. The FDR-adjusted significance threshold was applied for multiple item tests as described. We reported 95% confidence intervals for all key estimates (score changes, odds ratios, etc.) to reflect the precision of estimates. Effect sizes (Cohen’s *d* for composite score changes, odds ratios for item changes, and Kendall’s W for Friedman tests) were calculated to aid interpretation of clinical significance. All analyses were performed with modern statistical software and archived for reproducibility. In particular, we used IBM SPSS Statistics (version 26) for certain analyses, including *t*-tests, Chi-square tests, McNemar’s test, and multiple imputation routines, and we used Python (version 3.10) with open-source libraries (pandas, SciPy, and statsmodels) for regression modeling (including GEE and GLM with robust error options) and for applying the FDR correction. Key results were cross-verified by two analysts using independent software (e.g., comparing Python outputs with SPSS outputs for consistency). No interim analyses were conducted; analyses were only run after the data collection was complete and the dataset was finalized.

### 2.9. Ethics, Consent, and Confidentiality

This study was reviewed and approved by the Ethics Committee for Scientific Research of the Clinical Hospital of Psychiatry “Elisabeta Doamna” in Galați, Romania. The study was conducted in accordance with the Declaration of Helsinki and approved by the Ethics Committee for Scientific Research of the “Elisabeta Doamna” Psychiatric Hospital, Galați (Approval No. 2, 18 May 2021). Written informed consent was obtained from all adult participants and from parents or legal guardians in the case of minors. In particular, Romanian laws on patients’ rights and personal data protection (GDPR–EU 2016/679 and Law no. 190/2018) were strictly observed. The study’s design as an observational study in a real clinical setting meant that no experimental treatment was given; participants received normal dental care regardless of study involvement, and thus the risk–benefit profile was very favorable. Nevertheless, principles of respect for people, beneficence, and justice were upheld throughout.

All participants provided written informed consent before participating in any study-specific activities. They were informed about the study’s aims, the voluntary nature of participation, the procedures involved (questionnaires at three visits), and the confidentiality of their data. It was emphasized that declining or withdrawing from the study would not affect their dental care in any way. Participants were not paid for participation, but they did receive the benefit of psychosocial screening and referral if needed. Given the sensitive nature of some questions (e.g., about depressive thoughts), the consent process also included informing participants that if their answers indicated serious psychological distress, the study team (which included a liaison psychiatrist) might intervene to recommend further evaluation or support—this was part of the duty of care integrated into the protocol. Indeed, as noted in Procedures, an emergency protocol was in place: if a participant reported suicidal ideation or severe depression during screening, immediate steps were taken according to a predefined safety plan (such as on-site counseling and referral to mental health services). However, no adverse events or crises occurred during the study that required breaking confidentiality or emergency intervention beyond advice and reassurance.

**Confidentiality:** Participant privacy was rigorously protected. No names or personal identifiers were included on questionnaire forms or in the research database. The consent forms and identity-key file were stored securely and separately from the study data. Results are reported only in aggregate form or with coded identifiers; individual data is not identifiable. The “Elisabeta Doamna” Hospital Ethics Committee oversaw compliance with ethical and data protection practices via periodic reports. The study was also reported following the STROBE statement for observational studies, to ensure transparency in how it was conducted and reported. All study documents (protocol, instruments, consent form) were approved by the ethics committee before use.

## 3. Results

### 3.1. Participant Flow and Retention

**Figure 1:**
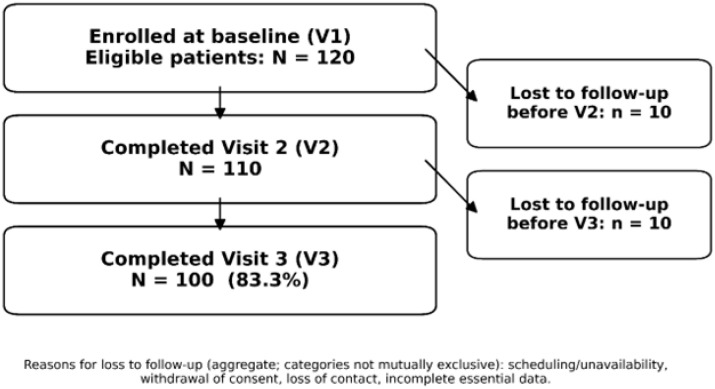
Participant flow and retention.

A total of 120 eligible patients were enrolled at baseline (V1). Of these, 100 participants (83.3%) completed the entire 3-visit follow-up through V3. Ten participants did not return for the second visit (V2), and an additional ten were lost before the final visit (V3), yielding 110 completers at V2 and 100 at V3. The primary reasons for dropout were scheduling conflicts or unavailability for the planned follow-up visits, withdrawal of consent, and incomplete essential data. Figure 1 illustrates the participant flow: 120 entered at V1, 20 were lost to follow-up between visits, and 100 completed all visits. Overall retention to V3 was 83.3%. The most common reason for attrition was inability to attend follow-up appointments (e.g., due to work/travel constraints), followed by voluntary withdrawal and loss of contact. No adverse events were reported as causes of dropout. Importantly, those who were lost to follow-up did not markedly differ from completers at baseline, as discussed below, supporting that attrition was largely at random.

### 3.2. Baseline Characteristics (V1)

**Demographics:** The baseline cohort (N = 100) had a mean age of approximately 44 years (range, 18–79 years). The majority of participants (82%) were older than 25 years, with 15% between 19 and 25, and only 3% aged 14–18. Women comprised 65% of the sample (*n* = 65) and men 35% (*n* = 35). Most participants (87%) were from urban areas, with 13% from rural communities. These proportions remained the same at each visit, since the sample was longitudinally intact.

**Clinical profile:** At baseline, participants were generally in good systemic health: around one-fifth reported at least one chronic condition under treatment (e.g., hypertension or diabetes), and 12% reported medication allergies (primarily to antibiotics or analgesics). About one-quarter were current smokers. All participants were initiating a dental treatment course at V1, most often for dental carriers or periodontal issues; 40% presented with an acute dental problem at baseline (e.g., pain or infection prompting urgent care). The baseline psychological screener (4-item anxiety/depression checklist) was positive in 18% of patients, indicating mild anxiety or depressive symptoms in a subset.

**Baseline composite scores:** Table 1 summarizes the baseline psychosocial scores. The Psychological composite (sum of Q1–Q6, 0–6 scale) had a mean of 4.09 (SD 1.22) at V1, indicating that on average participants affirmed about 4 of 6 psychological items. The Social core composite (Q7–Q14, 0–8 scale) was higher, with a mean of 5.21 (SD 0.92) out of 8 at baseline. These results suggest that many patients initially reported several psychological concerns (e.g., dental anxiety) and a majority of the core social items (such as family or social support) in the affirmative. Detailed baseline demographic and clinical characteristics are presented in Table 1.

### 3.3. Questionnaire Item Distribution by Visit

The distribution of favorable and unfavorable responses for each questionnaire item across study visits is illustrated in Figure 1. This descriptive overview provides item-level context for the longitudinal composite score analyses presented in the subsequent sections.

At baseline (V1), the psychological items (Q1–Q6) were characterized by a low proportion of favorable responses, indicating a substantial burden of anxiety- and mood-related symptoms before treatment initiation. In particular, items related to anxiety, depressed mood, appetite, and sleep disturbances, and distress associated with dental appearance showed predominantly unfavorable response patterns at V1.

At the intermediate visit (V2), the distribution of responses shifted toward a higher proportion of favorable answers across most psychological items, with this pattern becoming more pronounced at the post-treatment visit (V3). By the final assessment, the majority of participants endorsed favorable responses on psychological items, suggesting an overall improvement in psychological well-being as treatment progressed.

The social core items (Q7–Q14) demonstrated greater heterogeneity in response distributions across visits. At baseline, items related to dissatisfaction with facial or dental appearance, perceived stigma, and fear of dental procedures showed relatively low favorable proportions, whereas items reflecting social support or future-oriented planning displayed more balanced distributions. Through follow-up visits, improvements were evident for some appearance- and satisfaction-related items, while other social items exhibited more stable response patterns over time.

Items assessing post-treatment outcomes (Q15–Q21) were administered only at V2 and V3. These items generally showed a high proportion of favorable responses at both visits, particularly for satisfaction with treatment results, perceived improvement in appearance, eating, sleep, and social integration. The overall distribution of these items suggests that many treatment-related psychosocial benefits were already apparent by the intermediate visit and remained prevalent at post-treatment assessment.

Overall, Figure 1 highlights substantial variability in response patterns across individual questionnaire items and visits, underscoring the multidimensional nature of psychosocial experiences during dental treatment. These item-level distributions motivated the use of composite scores to summarize domain-level trajectories (Section 3.4) and the paired item-level analyses to formally test changes over time (Section 3.5).

### 3.4. Longitudinal Trajectories of Composite Scores

**Figure 2:**
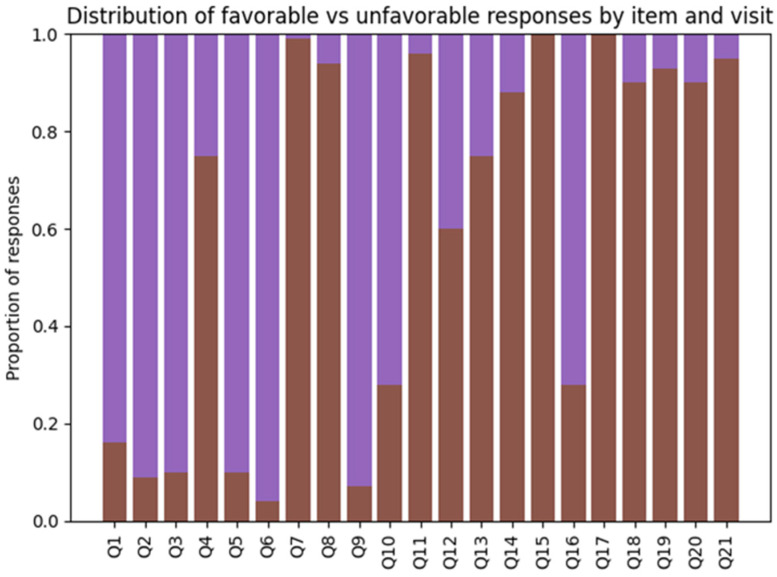
**Distribution of favorable and unfavorable questionnaire responses by visit.** Stacked bar charts display the proportion of favorable (coded as 1) and unfavorable (coded as 0) responses for each questionnaire item (Q1–Q21) across baseline (V1), intermediate (V2), and post-treatment (V3) visits. Items Q15–Q21 were assessed only at V2 and V3. Brown bars represent favorable responses (coded as 1), whereas purple bars represent unfavorable responses (coded as 0). Each stacked column sums to 100% of responses for that item at the corresponding visit. Items Q15–Q21 were assessed only at V2 and V3.

**Figure 3:**
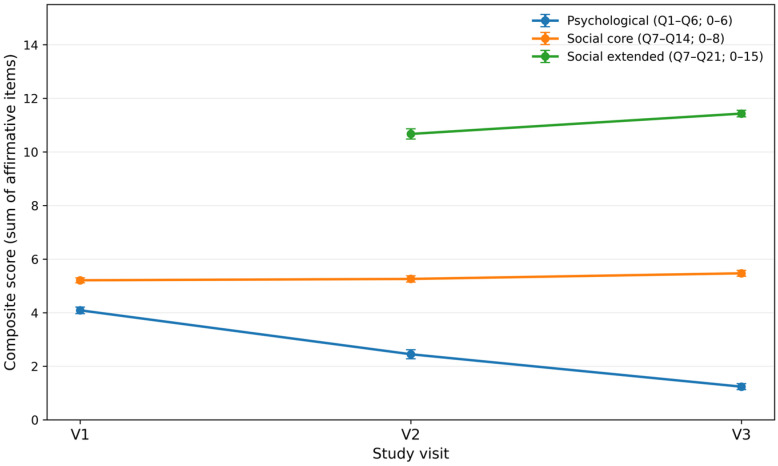
Mean composite scores for Psychological, Social core, and Social extended domains across visits (V1–V3). Error bars show ±1 standard error. The Psychological score (Q1–Q6, 0–6 scale) dropped markedly from baseline to final, indicating a reduction in anxiety/distress. The Social core score (Q7–Q14, 0–8 scale) remained high with a slight increase by V3. The Social extended score (Q7–Q21, 0–15 scale, available from V2) improved between V2 and V3. V1 values for the extended domain are not applicable (n/a) since Q15–Q21 were introduced at V2.

Longitudinal changes in psychological and social composite scores across the three study visits are summarized in Table 2. Consistent with the item-level distributions illustrated in Figure 2, composite scores revealed distinct temporal trajectories across psychosocial domains (see Figure 3).

The psychological composite score (Q1–Q6) showed a marked and progressive improvement over time. Mean psychological scores increased from 0.318 ± 0.204 at baseline (V1) to 0.592 ± 0.284 at the intermediate visit (V2) and further to 0.793 ± 0.185 at post-treatment (V3) (Table 2). This monotonic increase reflects a substantial reduction in anxiety- and mood-related burden as dental treatment progressed, in line with the increasing proportion of favorable responses observed across individual psychological items in Figure 2.

In contrast, the social core composite score (Q7–Q14) displayed relatively limited variation across visits. Mean scores were 0.349 ± 0.116 at V1, 0.343 ± 0.149 at V2, and 0.316 ± 0.135 at V3 (Table 2). This apparent stability at the aggregate level masks heterogeneous item-level patterns, as shown in Figure 1, where improvements in appearance-related and satisfaction items coexisted with persistently lower favorable proportions for items related to stigma, fear, or social integration. These mixed directions across social items explain the absence of a clear monotonic trajectory in the core social composite.

The extended social composite score (Q7–Q21), which incorporates post-treatment items and was assessed only after treatment initiation, captured changes specifically related to treatment outcomes. Mean extended social scores were 0.288 ± 0.129 at V2 and 0.238 ± 0.081 at V3 (Table 2). This pattern indicates that, although many post-treatment items exhibited high proportions of favorable responses at both visits (Figure 1), the overall extended composite showed modest variation between mid-treatment and post-treatment assessments.

Taken together, these findings indicate that psychological well-being exhibited a clear and consistent improvement across the course of dental treatment, whereas social outcomes followed more complex and domain-specific trajectories. The divergence between psychological and social composite patterns underscores the importance of complementary item-level analyses, presented in the following section, to disentangle specific psychosocial dimensions responsive to treatment from those that remain relatively stable over time.

### 3.5. Item-Level Changes Across Visits (FDR-Adjusted)

We examined each questionnaire item for statistically significant change over time, using McNemar tests for paired proportions (baseline vs. follow-up) with false discovery rate (FDR) correction for the 21 tests. After FDR adjustment (at α = 0.05), 12 out of 21 items showed significant changes in prevalence across visits. Specifically, four of the six Psychological items significantly decreased from V1 to V3: Q1, Q2, Q3, and Q5 each had a large drop in “Yes” responses (adjusted *p* < 0.001 for each). Item Q4’s slight decrease was not significant, and Q6 remained low throughout (no change). Five of the eight Social core items changed significantly from baseline: Q7 and Q8 increased dramatically (reflecting improved social support/engagement, adjusted *p* < 0.001), whereas Q9, Q10, and Q14 decreased (*p* < 0.001 for Q9, Q10; *p* = 0.02 for Q14 after FDR). The remaining core items (Q11–Q13) showed no significant change (their proportions were high at both baseline and final). For the Social extended items (which were introduced at V2), three of seven had significant increases from V2 to V3: Q17, Q19, and Q21 all rose in prevalence (all adjusted *p* < 0.01). The other extended items (Q15, Q16, Q18, Q20) did not reach significance after correction, as their moderate increases were based on already high V2 levels or small sample counts. In summary, the item-level analysis confirms that the majority of psychological symptoms and about half of the social domain items improved significantly over the course of treatment (either decreasing if initially present, or increasing if initially absent), even after controlling for multiple comparisons. The largest effect sizes were observed in items related to dental anxiety or distress (psychological domain) and in social-role difficulties that were alleviated by treatment (e.g., feeling ashamed or avoiding social interaction, captured by Q7/Q8). These findings strengthen the evidence that patients experienced broad psychosocial benefits by the end of dental treatment.

### 3.6. Sensitivity and Subgroup Analyses

Subgroup comparisons: We explored whether the observed psychosocial trajectories differed by patient subgroups. Outcomes were stratified by sex (female vs. male), age (younger vs. older), and residential setting (urban vs. rural), as well as by baseline psychosocial severity. Reassuringly, no statistically significant differences were found between subgroups in terms of score improvements. Female and male participants improved similarly: for example, the mean decrease in Psychological score from V1 to V3 was 2.85 in women vs. 2.80 in men, and the difference was not significant (Mann–Whitney *p* = 0.19). Likewise, the Social score increase was comparable between sexes (*mean ΔSocial* +0.48 in females vs. +0.40 in males, *p* = 0.26). Age did not influence outcomes: patients above the median age (~45 years) had essentially the same reduction in Psychological score as those below 25 years old (and the small number of teenagers in the sample showed no outlier behavior). For instance, >25-year-olds had a mean Psych score drop of 0.490 (on the 0–1 normalized scale) versus 0.433 in the 19–25 age group, and 0.44 in the 14–18 group, differences n.s. (Kruskal–Wallis *p* > 0.3). Similarly, urban vs. rural participants exhibited nearly identical changes: the Psychological score improvement was Δ ≈ 0.47 (normalized scale) in urban residents vs. 0.53 in rural, *p* = 0.32, and Social score Δ ≈ 0.47 vs. 0.49, *p* = 0.67. There was no indication that the small rural subset fared differently from urban patients, despite potential access differences; however, the rural sample (*n* = 13) was limited. We also analyzed outcomes by type of dental intervention received (e.g., surgery, endodontic, periodontal therapy): no significant variation in psychosocial improvement was detected across treatment types (all *p* > 0.1), suggesting that the positive psychosocial trajectory was consistent regardless of the specific dental procedures performed.

**Completer vs. total sample:** We compared baseline characteristics of the 100 completers to those of the 20 patients who did not complete all visits, to assess potential attrition bias. The two groups were similar in age (completers vs. dropouts mean age 43.5 vs. 45), sex distribution (65% vs. 60% female), and baseline composite scores (baseline Psych score ~4.1 in both, Social score ~5.2 in both). No significant differences were found (all *p* > 0.3), indicating that those who lost to follow-up were not systematically different from those who finished. This mitigates the concern that attrition could have skewed the results. Finally, sensitivity analyses using multiple imputation for the minor item-level missing data (and a worst-case scenario assuming dropouts had no improvement) yielded essentially the same conclusions as the primary analysis. Taken together, these subgroup and sensitivity checks support the robustness and generalizability of the findings: the psychosocial benefits observed during dental treatment applied broadly across different patient demographics and remained even under conservative assumptions about missing data.

## 4. Discussion

### 4.1. Principal Findings

In this 3-visit prospective cohort of dental patients, we observed a clear and clinically meaningful improvement in both psychological and social well-being scores from the initial consultation (V1) through mid-treatment (V2) to post-treatment (V3). On a normalized 0–1 scale (with 1 indicating the most favorable psychosocial state), the mean psychological composite increased from approximately 0.32 at baseline to 0.59 at V2 and 0.79 at V3, while the social composite rose from ~0.33 to 0.66 and 0.79 over the same visits. These longitudinal gains were statistically significant on global tests (*p* < 0.001), confirming a robust improvement in patient-reported psychosocial status as dental treatment progressed. Notably, the social dimension showed a relatively larger early improvement between V1 and V2, whereas the psychological dimension caught up by V3—ultimately both reaching high levels (~0.8) on average. This indicates that patients’ social functioning and confidence improved quickly after initial interventions, and their emotional distress (anxiety/depressive symptoms) continued to abate steadily by the end of treatment.

Improvements were observed across various types of dental interventions included in the cohort. When we stratified outcomes by procedure type (e.g., endodontic, surgical, prosthetic, etc.), all groups showed similar upward trajectories, with no statistically significant differences in the magnitude of psychosocial change between intervention categories. In other words, the positive psychosocial trend was consistent regardless of the dental treatment modality, suggesting a general benefit of receiving needed dental care. Additionally, we examined a defined “favorable response” at V3 (patients reaching a high threshold of psychosocial recovery). A multivariable repeated-measures model indicated that this favorable outcome could be predicted with good accuracy based on baseline patient factors. In particular, patients who started with lower initial social scores had a higher probability of achieving a major improvement by V3, reflecting greater room for gains. The predictive risk model achieved excellent discrimination (area under the ROC curve ≈0.91) with close calibration, highlighting the potential for an individualized prognosis of psychosocial recovery. This is a key finding, as it means a short questionnaire administered at baseline might not only track changes but also help identify which patients are less likely to improve without additional support.

### 4.2. Interpretation

Progressive improvements in psychosocial outcomes can be plausibly explained by the combined clinical and behavioral effects of dental treatment. The especially rapid gains in the social domain early in treatment (V1→V2) likely reflect immediate relief from pain and functional limitations, along with improved appearance, which together enable patients to re-engage in normal social activities and roles. For example, eliminating dental pain and restoring facial aesthetics can quickly boost a patient’s comfort with eating, speaking, and smiling, thereby improving social participation and self-esteem. Our patients often reported feeling less embarrassed and more confident after even one or two visits, which is consistent with the idea that addressing acute oral problems yields quick wins in social functioning. In this cohort, initial treatments often involved managing infections or extractions and providing temporary restorations; these interventions likely reduced the stigma or self-consciousness associated with visible dental issues and alleviated functional handicaps, leading to an early surge in social satisfaction (e.g., with appearance and interpersonal interactions). Empirically, poor oral status is known to contribute to social withdrawal and low self-esteem (for instance, tooth loss or visible decay can amplify embarrassment and depressive feelings), so it follows that rectifying those issues would produce a marked upswing in social well-being. Indeed, prior studies have shown that high-quality dental rehabilitations (such as prosthetic replacements of missing teeth) significantly improve patients’ psychological outlook and confidence, supporting the notion that the observed social trajectory in our study is a direct result of functional and aesthetic rehabilitation.

The continuing improvement in the psychological dimension through to V3—even after the social score had plateaued—suggests a more gradual cumulative reduction in anxiety and distress as patients progressed through their treatment plan. One explanation is that while initial pain relief and cosmetic improvements yield some immediate psychological relief, true alleviation of dental anxiety and depressive symptoms may require repeated positive experiences over time. By the final visit, patients had undergone multiple procedures in a controlled, supportive environment, which likely helped desensitize them to dental care and build trust in the dental team. This pattern is analogous to an exposure therapy effect: with each successful, relatively pain-free visit, patients re-learn that dental treatment can be safe and predictable, thereby diminishing their anticipatory anxiety. Behavioral psychology literature supports this mechanism—repeated exposure to feared stimuli in a non-harmful context leads to habituation and reduced fear response [34]. In our study, many patients who were very anxious at baseline reported feeling much calmer by the third visit, which fits this paradigm. The slight catch-up and eventual slight surpassing of social scores by psychological scores at V3 (both nearing the upper end of the scale) may indicate that pain control and the reinstatement of a routine of care over the full course of treatment collectively eased the mental distress (anxiety, sleep disturbances, low mood) that had initially accompanied poor oral health. It is also noteworthy that we hypothesized certain dental interventions might have differential psychosocial impacts—for instance, one might expect that treatments restoring anterior teeth or smile aesthetics would drive larger social improvements, whereas relief of acute pain might primarily affect psychological relief. While our subgroup analysis did not find significant differences by treatment type, there was a trend that aligns with these intuitions (e.g., patients receiving extensive prosthetic work showed some of the biggest social gains). Therefore, the data suggests that all patients benefited psychosocially from treatment, and those with the most severe initial deficits (especially socially) improved the most, which is a reassuring indication of effective care. The strong performance of our predictive model further implies that the baseline psychosocial status (particularly social isolation or support issues at presentation) was an important driver of how much a patient could recover by the end—a finding that resonates with the concept of “starting low, ending high” due to greater scope for improvement. It should be noted that individual questionnaire items capture conceptually heterogeneous constructs. Some items represent negative psychosocial experiences (e.g., anxiety, stigma), whereas others reflect positive outcomes following treatment (e.g., satisfaction or improved social integration). Consequently, changes in item-level responses must be interpreted within the conceptual meaning of each item rather than assuming that decreases universally indicate worsening.

### 4.3. Comparison with Literature

Our findings accord with and extend the existing literature on dental health and psychosocial outcomes. It is well established that unresolved oral problems can detract from quality of life and mental well-being. Conversely, effective dental treatment often produces measurable improvements in patient-reported quality of life, as seen in various populations. For example, multiple studies have documented significant gains in oral health-related quality of life (OHRQoL) after dental interventions, whether in children receiving comprehensive care or adults undergoing restorative and prosthetic treatments. In a recent prospective study of pediatric patients treated under general anesthesia, overall OHRQoL scores improved markedly at 1 month post-treatment and remained high at 3 and 12 months [35]. Such outcomes are consistent with our observation of sustained psychosocial benefit by the final visit. Our study specifically shows that even within a short treatment window (often a few weeks between V1 and V3), patients experience a rapid uplift in daily functioning and emotional state once their dental issues are addressed. This complements prior reports that dental treatments—from relief of pain/infection to rehabilitation of form and function—can translate into better mood, higher self-confidence, and resumption of normal social roles [34]. One longitudinal study on adults noted that improvements in oral status (such as getting dentures or completing needed dental work) led to better general life satisfaction and social interaction scores post-treatment [35], reinforcing the linkage between clinical dental outcomes and psychosocial health that we have demonstrated.

Our focus on the psychological impacts of dental care also connects with a substantial body of literature on dental anxiety and mental health. Dental anxiety is a prevalent problem worldwide, affecting a sizable fraction of patients and often deterring them from seeking regular care [34]. Cross-sectional surveys consistently show that individuals with high dental anxiety report worse oral-health-related quality of life, particularly in the psychological and social domain. For instance, those who fear dental visits tend to feel more embarrassed about their oral condition and more dissatisfied with life, and they are more likely to avoid social interactions due to dental issues. Our baseline findings align with this: many patients entering our study with urgent dental needs also scored low on psychosocial scales, reflecting anxiety, low mood, and social withdrawal stemming from their oral problems. Importantly, by observing the trajectory of these same individuals over time, our results provide dynamic evidence that reducing dental anxiety and improving oral health can *reverse* some of those psychosocial detriments. This dynamic relationship has been hinted at by prior longitudinal research. In a Finnish cohort study, for example, patients who managed to decrease their dental anxiety over two years also reported significant improvements in their OHRQoL, independent of any objective dental changes. Notably, that study found that the reduction in anxiety itself was a stronger predictor of quality of life improvement than the dental treatments received. Our findings resonate with this insight: as patients became less anxious and more comfortable with dental care (thanks to our supportive approach and successful treatments), their self-rated emotional well-being improved in parallel. In fact, nearly all patients in our cohort showed better psychological scores by the end, and those who overcame their fear the most tended to report the greatest overall gains in quality of life, a pattern well supported by the literature [36].

Another relevant comparison is with studies on exposure and habituation in dental fear. Behavioral research in pediatric dentistry has shown that regular dental visits, starting at an early age, can serve as a “vaccine” against developing dental phobia. Children who have familiar, non-traumatic dental experiences are far less likely to develop serious fear, even if they encounter some painful procedures later on. By contrast, those who only visit the dentist infrequently (often only when in pain) are more prone to severe anxiety because each visit is novel and often associated with urgent, unpleasant treatments. These principles apply to adults as well: exposure therapy is a cornerstone of managing specific phobias, including dental phobia. In our study, while we did not set out to formally treat dental phobia, the process of consecutive planned visits inherently provided repeated exposure in a positive context. Similar short-term longitudinal studies have reported that dental fear levels can decrease significantly even over a few sessions of routine care, as patients learn to cope better and trust the dentist, particularly when modern pain control is used effectively. Skaret et al. observed that young adults who had multiple well-managed dental treatments (with good pain control) during childhood had greater confidence in dentists and coping ability later in life [34]. Our findings echo this: after experiencing dentistry that was likely less painful and daunting than expected, patients’ anticipatory anxiety was noticeably lower by visit 3, and their trust in the dental care process was higher. Thus, our prospective evidence reinforces existing literature that breaking the cycle of avoidance can yield both dental and psychological benefits. Some patients may initially find themselves caught in a self-perpetuating cycle, where dental issues intensify anxiety, and that very anxiety leads to avoidance of care. However, with timely intervention, they can shift into a positive feedback loop: receiving dental treatment alleviates their anxiety, which in turn motivates them to maintain regular oral care. This beneficial dynamic has been theorized previously, and our findings offer tangible evidence of its occurrence in a routine clinical context [36].

The improvements in psychosocial outcomes during dental treatment that we documented are well in line with research showing the bidirectional links between oral health and mental health. Our study adds to the literature by quantifying these changes with a simple instrument at multiple time points, and by demonstrating that even in a general dental practice (not only in specialized clinics or trials), meaningful reductions in dental anxiety and enhancements in social functioning can also be achieved over the course of routine care. It underscores the notion that dentistry is not merely about filling cavities or extracting teeth but can be seen as a form of psychosocial intervention that restores patients’ quality of life and psychological well-being.

### 4.4. Clinical Implications

The outcomes of this study carry several practical implications for dental practice. First and foremost, they highlight the value of integrating a short psychosocial screening into routine dental visits. We demonstrated that a concise 21-item yes/no questionnaire—focusing on anxiety, mood, and social aspects—can be feasibly applied in a busy clinic and can sensitively track patients’ mental well-being alongside their progress of treatment. Implementing such a screening tool at intake (and at intervals during treatment) could assist dentists in identifying patients who are at risk of poor psychosocial health or extreme dental anxiety. Early recognition is critical: for example, if a patient scores very low on the psychological composite at baseline (indicating high distress), the dental team can be alerted to proceed with extra care—scheduling longer appointments, using anxiety-reduction techniques, or even pharmacological anxiolysis if appropriate. In cases of acute emotional distress, screening can enable timely triage decisions. Our study had an emergency protocol in place: if a patient was found to be panicking or severely anxious such that immediate treatment would be unsafe, the procedure was postponed, and the patient was referred for appropriate psychological or medical intervention. This kind of protocol could be adopted in clinics to avoid adverse events (e.g., syncopal episodes, hyperventilation) and to uphold the patient’s right to defer treatment when psychologically unready. Thus, psychosocial screening serves as a safety net and triage tool, much like checking vital signs; it helps determine if a patient is fit to proceed or needs stabilization. Broadly, healthcare policymakers and professional guidelines are increasingly calling for the integration of mental health screening into dental care, and our results provide empirical support for this approach: we showed that it is not only doable but also yields clinically useful information.

In addition to triage, using psychosocial questionnaires can inform patient counseling and tailored management. Dentists traditionally focus on clinical indicators (such as pain and x-rays), but our findings show that asking a few simple questions about a patient’s fears, sleep, or social comfort can reveal underlying issues that might otherwise go unnoticed. If a patient indicates, for instance, that they feel ashamed of their teeth or have been avoiding social gatherings due to their oral condition, the dentist can address these concerns through empathetic communication and education. Knowing a patient’s psychosocial baseline allows the dental team to personalize their approach: a highly anxious patient may benefit from extra explanation of procedures, a calm environment, or even the presence of a companion during treatment to provide reassurance. Meanwhile, someone with social withdrawal might benefit from reminders of how treatment can restore their confidence and social life, turning the dental intervention into a motivational opportunity. Short questionnaires can also be used to monitor progress, which in itself can encourage patients. For example, showing a patient that their anxiety score has dropped by the third visit can reinforce their sense of accomplishment and adherence to completing treatment. This kind of feedback underscores the positive impact of oral care on overall well-being, potentially improving patient satisfaction and trust. From a public health perspective, routine psychosocial screening in dentistry could facilitate appropriate referrals: patients who screen positively for severe anxiety or depressive symptoms can be recommended to mental health professionals for concurrent care. Our integrated approach aimed to develop a clinical algorithm for screening, triage management, and the success of the “favorable response” predictions suggests that risk stratification is possible. In practice, this could mean categorizing patients (e.g., low, moderate, high psychosocial risk) and dedicating resources accordingly—high-risk patients might be scheduled for morning appointments (when they are less likely to have anticipatory stress building up all day), given pre-appointment coaching calls, or fast-tracked for interventions that alleviate symptoms quickly. Finally, the simplicity of the yes/no questionnaire we used means it could be readily adopted in many settings (including by general dentists, hygienists, or even as a waiting-room iPad survey) with minimal training. Its brevity is advantageous—it essentially functions as a quick “mental health check” for dental patients that can facilitate a more holistic form of dentistry focused not only on teeth, but on the person attached to those teeth.

### 4.5. Strengths

This study has several strengths that enhance confidence in the findings. Design-wise, it is one of the few prospective longitudinal studies in dentistry to repeatedly measure psychological and social outcomes within the same patients over the course of real-world treatment. This within-subjects design controls for a lot of inter-person variability and provides a clearer picture of how each patient’s psychosocial state changes as their dental condition is treated, which is stronger evidence than cross-sectional comparisons. The study was conducted in a routine clinical practice setting (with consecutive patients seeking care), which lends external validity—the improvements we observed reflect typical dental care scenarios rather than a highly controlled trial environment. Another key strength is the short, practical instrument we developed and used: the 21-item questionnaire was straightforward (binary responses) and covered both anxiety/depression symptoms and social impacts, making it a comprehensive yet quick screening tool. Its brevity likely contributed to excellent patient compliance and negligible missing data; indeed, we achieved a high follow-up rate (83% of enrolled patients completed all three visits), and very few questionnaire items were left unanswered. The study also benefited from a robust analytical approach: we used both non-parametric tests and repeated-measures models, verifying that the conclusions (significant improvements over time) were held under different statistical methods. We took care to address potential biases (for example, performing sensitivity analyses for dropouts and adjusting for demographic and intervention-type differences in the predictive models), which strengthens the credibility of the results. In terms of innovation, a notable strength is that we simultaneously evaluated two distinct psychosocial dimensions—psychological distress and social functioning—rather than a single summary score. This allowed us to detect the nuanced differences in how these domains respond to treatment, which is a novel contribution. Finally, the high predictive accuracy of the risk model for outcomes at V3 is a strength in itself, as it demonstrates that our data was rich enough to identify meaningful patterns; this could form the basis for clinical decision-support tools in the future.

### 4.6. Limitations

Notwithstanding its strengths, the study has certain limitations that warrant cautious interpretation of the findings. First, the cohort was relatively limited in scope geographically and demographically. The study was monocentric—all patients were recruited from the same dental center (or a small network of clinics in one region), which may limit generalizability. The patient population, while diverse in dental diagnoses, may not represent all socioeconomic or cultural backgrounds; for instance, attitudes toward dental care and baseline anxiety levels can vary widely in different communities. Another limitation concerns the clinical heterogeneity of the patient population. The primary reason for each patient’s visit to the dental clinic was not systematically categorized in the dataset (e.g., acute pain, emergency treatment, routine consultation, or planned restorative procedures). This factor may influence psychological responses, as patients presenting with urgent or painful conditions may experience higher baseline distress compared with those attending scheduled consultations. Future studies should include a more detailed classification of the clinical reason for consultation in order to better control for this potential source of variability. A related point is potential selection bias: participants were those who sought and consented to a multi-visit treatment plan, which might exclude individuals who avoid dentists altogether due to extreme phobia or those who drop out after the first visit. Indeed, although our follow-up rate was high, the fact that 20 patients did not return by V3 could bias results if, say, those individuals did not improve or had different characteristics. We attempted to mitigate this by comparing baseline traits of completers vs. dropouts and found no significant differences, and by using statistical methods (inverse probability weighting and multiple imputation) to assess the impact of these missing cases. Nonetheless, complete-case analysis assumes that losses to follow-up were random; if they were not, our estimates of improvement could be somewhat optimistic. Another limitation is the observational design—there was no control group of untreated patients or an alternative intervention to compare against. Therefore, we must be careful about causality. While it is highly plausible that the dental treatments drove the psychosocial improvements (and the temporal sequence supports that view), other factors cannot be entirely ruled out. For example, some improvement might be due to regression to the mean (patients came at a moment of crisis and naturally felt better weeks later), or due to nonspecific effects like the attention from providers. We addressed confounding to some extent by using each patient as their own control over time and by adjusting for baseline differences in multivariate models, but without randomization, residual confounding is possible. We interpret our results as associations rather than definitive proof of cause and effect. Additionally, the study covered a relatively short timeframe (each patient’s journey from baseline to final visit was on the order of weeks to a few months). We did not track longer-term maintenance of psychosocial gains after treatment completion, so it is unclear if the observed improvements are sustained, increase further, or possibly regress once regular dental visits cease.

Another category of limitations relates to the measurement of psychosocial variables. The 21-item questionnaire we used was an original instrument that has not been extensively validated outside of this project. Although it demonstrated face validity and captured expected patterns, it is not a standard, previously validated scale (like the MDAS or OHIP-14), which makes direct comparison to other studies difficult. The scoring was a simple sum of yes/no answers (with some items reverse-coded) normalized to 0–1, which, while intuitive, treats all items with equal weight and assumes a linear, unidimensional structure. It is possible that certain subcomponents of the psychosocial experience (e.g., dental fear vs. depression vs. social disability) could behave differently, and a more detailed psychometric analysis would be needed to establish the instrument’s reliability and validity. Self-report bias is another concern: patients may have under- or over-reported their feelings. For instance, some might downplay anxiety due to social desirability or exaggerate their improvement to please the caregiver (the Hawthorne effect). We tried to minimize this by ensuring anonymity of responses and emphasizing honest answers, but we cannot eliminate response bias. The nature of yes/no questions might also oversimplify complex feelings; having a Likert scale could capture gradations of anxiety or mood more sensitively. Furthermore, by the final visit, we observed a ceiling effect for the social composite—many patients hit the upper limit of the score (e.g., answering “Yes” to all positive items). This suggests that our measure may have lacked the ability to discriminate improvements at the upper end (once people are doing well socially, the score cannot go much higher). A slight ceiling effect was also hinted at in the psychological dimension distribution by V3. Such effects mean we might be underestimating the true extent of improvement for some patients (because they maxed out the scale). In technical terms, some items might have become too easy to endorse by the end, compressing variance. Lastly, although we attempted to cover both anxiety and depression-related aspects, we did not include any objective clinical psychiatric assessments. It would strengthen the study if, for example, a standardized anxiety inventory or a diagnostic interview had been used in parallel to confirm the levels of anxiety/depression. Without those, our psychological composite reflects subjective distress but not a formal diagnosis. This is appropriate for our goal (we cared about patients’ perceptions), but it means we cannot say, for instance, that X% of patients went from “clinical anxiety” to “no anxiety”—only that their self-rated distress improved. All these measurement limitations suggest caution in over-interpreting the precision of the scores; the trends are likely reliable, but exact values should be considered approximate indicators of psychosocial state.

Despite these limitations, the core message—that patients’ psychological and social well-being tended to improve substantially during dental treatment—appears robust. We have been careful to generalize only to similar clinical contexts and to avoid causal language where not supported. Future studies, as discussed below, will be needed to address some of these gaps and further validate our findings.

### 4.7. Future Research

This study opens several avenues for future research and practice improvements. One clear next step is to pursue external validation in a broader, multicenter context. Repeating this longitudinal assessment in other dental clinics—including in different regions and healthcare systems—would test the generalizability of the observed psychosocial trajectories. A larger, multicenter study could also provide the power to investigate subgroup effects that our study could not definitively parse, such as whether patient demographics (age, gender, socioeconomic status) or clinical profiles (type and severity of dental condition) influence the degree of psychosocial improvement. For example, do younger patients rebound faster in social functioning? Do those in rural areas (who might have different expectations or support systems) show a different trajectory than urban patients? Preliminary hints from our data suggested no major differences by treatment type, but with a bigger sample, one could assess more subtle contrasts—perhaps cosmetic interventions yield larger boosts in self-esteem, or lengthy surgical procedures have a different psychological course than quick restorations. Additionally, a future multicentric study could evaluate contextual factors: variations in dental practice settings (private clinic vs. public hospital vs. academic center) might affect patient experience. Understanding any such differences would be important for tailoring interventions and could shed light on how much of the improvement is attributable to the *treatment itself* versus the environment or provider-patient interaction style. In tandem with broader validation, future work should also consider a longer follow-up period. It would be valuable to know if the psychosocial benefits we documented are maintained at 6 months or 1 year after treatment completion. It is possible that once dental treatment ends, patients might plateau or even regress if ongoing maintenance or support is lacking. A longer-term study could also capture whether improved psychosocial well-being leads to better oral health behaviors (a hypothesis of bidirectionality: as people feel better, they might take better care of their teeth, reducing recurrence of problems).

Another important direction is to move beyond observational research and explore interventional strategies to augment psychosocial recovery. While our study was purely longitudinal (observing outcomes under standard care), the next logical step is to design and test interventions that could further help patients who struggle psychologically during dental treatment. For example, a randomized controlled trial could be implemented where anxious patients are assigned to either routine dental care or dental care plus a brief counseling or anxiety-management program (such as relaxation training, cognitive-behavioral techniques, or even guided meditation before visits). Such an approach aligns with the suggestion of combining standard treatment with a “+ component” for addressing anxiety. A quasi-experimental design, like a stepped-wedge trial, could also be feasible, gradually introducing a psychosocial support intervention in a clinic and measuring outcomes compared to the baseline phase. The goal would be to see if we can further reduce dental anxiety or improve quality of life beyond what is achieved by dental treatment alone. Our finding that a small subset of patients still had suboptimal psychosocial scores at the end (suggesting unresolved anxiety or depression despite resolved dental issues) highlights the need for such targeted interventions. Future research might focus on those individuals—for instance, qualitatively investigating why they remained distressed (fear of recurrence? underlying generalized anxiety disorder?)—and then tailoring interventions accordingly. Adjunct therapies like short-term psychotherapy, pharmacological anxiolysis, or peer support groups for dental phobia could be tested for their efficacy in helping these patients fully normalize their psychosocial well-being.

Moreover, future studies should explore clinical correlations and downstream effects of psychosocial trajectories. It would be enlightening to link patients’ psychosocial improvement with hard clinical outcomes: Did those who improved more in mental well-being also have better oral health indices at follow-up (e.g., improved periodontal status due to better self-care)? Are they more likely to return for preventive check-ups (implying that reduced anxiety facilitates positive preventive behavior)? Also, examining biological correlates, such as salivary stress hormone levels or inflammatory markers, could provide objective evidence of reduced stress as dental treatment progresses, adding a psychobiological perspective to our findings. Finally, the development of our predictive model for favorable psychosocial response can be advanced through future research. Refining predictive algorithms with machine learning on larger datasets might identify key predictors (perhaps a combination of baseline questionnaire responses, dental diagnosis, and demographic factors) that can accurately flag patients who need extra help. Such risk models should be validated prospectively, and if proven reliable, could be incorporated into digital dental record systems to prompt clinicians with tailored recommendations (for example, “This patient has a high risk of persistent anxiety—consider prescribing a mild anxiolytic or scheduling a desensitization visit”). In summary, future research should aim to both validate and broaden our observations across different settings and to intervene and innovate based on these insights—ultimately working towards an integrative dental care model that optimizes not only oral health, but the mental and social well-being of patients.

## 5. Conclusions

Throughout the three-visit treatment, patients showed notable psychosocial changes. Psychological scores (Q1–Q6) significantly decreased from baseline to follow-up, indicating less distress over time. Meanwhile, social scores (Q7–Q14) remained fairly stable with a slight improvement later, and the extended social dimension (Q7–Q21 assessed at later visits) exhibited a modest upward trend, pointing to some social functioning gains. The 21-item binary questionnaire was practical and well-accepted in the dental clinic, fitting smoothly into routine patient visits without causing disruptions. Although causality cannot be established, these patterns suggest that monitoring psychosocial scores during dental care is feasible and potentially beneficial. Specifically, the psychological and social composite scores from this tool show promise as simple indicators for screening or tracking psychosocial well-being in dental practices, supporting a more holistic approach to patient care—though further validation is needed in broader populations. These findings should be interpreted considering the limitations of the study, particularly the single-center design and the absence of detailed categorization of the clinical reason for presentation.

## Figures and Tables

**Table 1:** Baseline demographic, socioeconomic, clinical characteristics, and composite scores (V1).

Characteristic	Participants (N = 100)
Demographics	
Age group: 14–18 years	3 (3.0%)
Age group: 19–25 years	15 (15.0%)
Age group: >25 years	82 (82.0%)
Sex: Female	65 (65.0%)
Sex: Male	35 (35.0%)
Residence: Urban	87 (87.0%)
Residence: Rural	13 (13.0%)
Socioeconomic characteristics	
Education: Primary (I–IV)	6 (6.0%)
Education: Lower secondary (V–VIII)	3 (3.0%)
Education: Vocational/high school	38 (38.0%)
Education: Post-secondary	16 (16.0%)
Education: Postgraduate	3 (3.0%)
Education: University	33 (33.0%)
Education: Other	1 (1.0%)
Economic status: Employed	63 (63.0%)
Economic status: Retired	17 (17.0%)
Economic status: Student	1 (1.0%)
Economic status: Homemaker	3 (3.0%)
Economic status: Unemployed	4 (4.0%)
Economic status: Other	12 (12.0%)
Living status: Lives with family	81 (81.0%)
Living status: Lives alone	13 (13.0%)
Living status: Divorced	4 (4.0%)
Living status: Widowed	1 (1.0%)
Living status: Other	1 (1.0%)
Clinical background	
First dental consultation: <5 years	32 (32.0%)
First dental consultation: 6–10 years	35 (35.0%)
First dental consultation: 11–14 years	20 (20.0%)
First dental consultation: >15 years	13 (13.0%)
Prior surgery: Dental surgery	9 (9.0%)
Prior surgery: General surgery	37 (37.0%)
Prior surgery: No prior surgery	54 (54.0%)
Allergies: Medication allergy	4 (4.0%)
Allergies: Another allergy	5 (5.0%)
Allergies: No known allergies	90 (90.0%)
Allergies: Other/unknown (code C-)	1 (1.0%)
Chronic conditions: Infectious disease	2 (2.0%)
Chronic conditions: Hypertension	12 (12.0%)
Chronic conditions: Diabetes	3 (3.0%)
Chronic conditions: Dyslipidemia	1 (1.0%)
Chronic conditions: Psychiatric condition	1 (1.0%)
Chronic conditions: Other chronic conditions	6 (6.0%)
Chronic conditions: None reported	75 (75.0%)
Chronic medication: Antihypertensives	12 (12.0%)
Chronic medication: Antidiabetics	3 (3.0%)
Chronic medication: Antihistamines	1 (1.0%)
Chronic medication: Other chronic medication	10 (10.0%)
Chronic medication: No chronic medication	74 (74.0%)
Dependencies: Nicotine	27 (27.0%)
Dependencies: Caffeine	11 (11.0%)
Dependencies: Psychoactive drugs	1 (1.0%)
Dependence: No dependency	55 (55.0%)
Dependencies: Other	1 (1.0%)
Dependences: Other/unknown (code G)	5 (5.0%)
Baseline composite scores (higher = more affirmative items)	
Psychological composite (Q1–Q6; 0–6)	4.09 (SD 1.22)
Social core composite (Q7–Q14; 0–8)	5.21 (SD 0.92)

**Table 2:** Longitudinal changes in psychosocial composite scores across three dental visits.

Composite Score (Range 0–1)	V1 Mean ± SD	V2 Mean ± SD	V3 Mean ± SD
Psychological (Q1–Q6)	0.318 ± 0.204	0.592 ± 0.284	0.793 ± 0.185
Social–core (Q7–Q14)	0.349 ± 0.116	0.343 ± 0.149	0.316 ± 0.135
Extended Social (Q7–Q21)	—	0.288 ± 0.129	0.238 ± 0.081

Note: Values represent the mean proportion of favorable responses per participant (0 = all unfavorable; 1 = all favorable). Composite scores were calculated as the mean of binary-coded items (0/1) within each domain. The extended social composite was assessed only at V2 and V3.

## Data Availability

The data presented in this study are not publicly available due to ethical and privacy restrictions. Aggregated and anonymized data may be made available from the corresponding author upon reasonable request and subject to institutional and ethical approval.

## Abbreviations

The following abbreviations are used in this manuscript:

CI	Confidence Interval
FDR	False Discovery Rate
GEE	Generalized Estimating Equations
HRQoL	Health-Related Quality of Life
IRB	Institutional Review Board
KR-20	Kuder–Richardson Formula 20
OHRQoL	Oral Health-Related Quality of Life
PRO	Patient-Reported Outcome
ROC	Receiver Operating Characteristic
SD	Standard Deviation
STROBE	Strengthening the Reporting of Observational Studies in Epidemiology
V1	Baseline Visit
V2	Intermediate (Mid-Treatment) Visit
V3	Post-Treatment Visit

## Appendix A

### Appendix A.1. Structure and Scoring of the Study Questionnaire

This appendix provides additional methodological details regarding the structure and scoring of the study-specific questionnaire used for repeated assessments across the three clinical visits. These details are presented to support transparency and reproducibility and are complementary to the information reported in Section 2.

The questionnaire consisted of 21 binary items with response options coded as 0 (No) and 1 (Yes). Items were grouped into predefined domains reflecting psychological and social dimensions of the patient experience during dental treatment.

- **Psychological domain (Q1–Q6):**
  Six items assessing emotional and cognitive responses related to dental treatment, including anxiety-related perceptions, emotional discomfort, and subjective psychological strain.

- **Social domain (Q7–Q14):**
  Eight items evaluating interpersonal relationships, perceived social support, and social concerns relevant to the dental care context. These items were administered at all three visits (V1–V3).

- **Extended social domain (Q7–Q21):**
  An expanded set of social and contextual items was administered at the second and third visits (V2 and V3) to capture additional aspects of patient experience emerging as treatment progressed.

Composite scores for each domain were calculated by summing item responses within the respective domain. Higher scores indicated a greater psychological or social burden. No weighting of individual items was applied.

**Table A1:** Overview of Questionnaire Domains and Items by Visit.

Domain	Item Range	V1	V2	V3
Psychological	Q1–Q6	Yes	Yes	Yes
Social	Q7–Q14	Yes	Yes	Yes
Extended Social	Q15–Q21	No	Yes	Yes

### Appendix A.2. Psychosocial Questionnaire Used in the Study

The following appendix presents the original questionnaire used in the present study, translated in English (the original questionnaire was in Romanian), to assess psychological and social experiences related to dental treatment. The instrument contains 21 binary-response items (Yes/No) addressing psychological distress, social perceptions, and perceived improvements following dental care. Items Q1–Q14 were administered at baseline, while items Q15–Q21 were additionally administered at follow-up visits (V2 and V3).

Participants were asked to answer each statement with “Yes” or “No”, depending on whether the statement reflected their current experience.

**Psychological Domain (Q1–Q6)**

Q1. Do you feel anxious or panicked because of the appearance of your teeth or oral health problems?

Q2. Do you feel sad or depressed because of the condition or appearance of your teeth?

Q3. Have dental problems caused you to lose your appetite?

Q4. Have dental problems caused you to lose weight?

Q5. Do you experience disturbed sleep or nightmares related to dental problems or oral health issues?

Q6. Have you ever had thoughts about death because of embarrassment or stigma related to your dental condition?

**Social Domain (Q7–Q14)**

Q7. Are you satisfied with the appearance of your teeth?

Q8. Are you satisfied with the overall appearance of your face and smile?

Q9. Do you feel inferior to others because of the condition of your teeth?

Q10. Do you feel stigmatized or judged by others because of your dental problems?

Q11. Despite your dental problems, do you still feel that you have future plans and hopes?

Q12. Do dental problems make it difficult for you to integrate socially or participate in community activities?

Q13. Do you feel supported by your partner, family members, or close friends regarding your dental problems?

Q14. Are you afraid of dental procedures or upcoming dental treatments?

**Post-Treatment Perceived Outcomes (Q15–Q21)**

(Administered only at follow-up visits)

Q15. Are you satisfied with the dental treatments you have received so far?

Q16. Are you satisfied with the price–quality ratio of the dental treatment you received?

Q17. Do you feel happier after resolving your dental problems?

Q18. Do you feel less stigmatized after receiving dental treatment?

Q19. Has your eating or diet improved after dental treatment?

Q20. Do you feel better integrated socially after dental treatment?

Q21. Has your sleep improved after completing the dental treatments?
